# Human T Lymphotropic Virus Type I (HTLV-I) Oncogenesis: Molecular Aspects of Virus and Host Interactions in Pathogenesis of Adult T cell Leukemia/Lymphoma (ATL)

**Published:** 2013-03

**Authors:** Sanaz Ahmadi Ghezeldasht, Abbas Shirdel, Mohammad Ali Assarehzadegan, Tahereh Hassannia, Hosian Rahimi, Rahele Miri, S. A. Rahim Rezaee

**Affiliations:** 1 Research Centre for HIV/AIDS, HTLV and Viral Hepatitis, Iranian Academic Centre for Education, Culture & Research (ACECR), Mashhad Branch, Mashhad, Iran; 2 Inflammation and Inflammatory diseases research Centre, Medical School, Mashhad University of Medical Science, Mashhad, Iran; 3 Department of Immunology, Faculty of Medicine, Ahvaz Jundishapur University of Medical Sciences, Ahvaz, Iran; 4 Internal Medicine Dept, Medical School, Arak University of Medical Sciences, Arak- Iran; 5 Immunology Research Centre, Mashhad University of Medical Sciences, Mashhad, Iran

**Keywords:** Adult T Cell Leukemia/Lymphoma, HTLV-I, Oncoviruses, Oncogenecity

## Abstract

The study of tumor viruses paves the way for understanding the mechanisms of virus pathogenesis, including those involved in establishing infection and dissemination in the host tumor affecting immune-compromised patients. The processes ranging from viral infection to progressing malignancy are slow and usually insufficient for establishment of transformed cells that develop cancer in only a minority of infected subjects. Therefore, viral infection is usually not the only cause of cancer, and further environmental and host factors, may be implicated.

HTLV-I, in particular, is considered as an oncovirus cause of lymphoproliferative disease such as adult T cell leukemia/lymphoma (ATL) and disturbs the immune responses which results in HTLV-I associated meylopathy/tropical spastic parapresis (HAM/TSP). HTLV-I infection causes ATL in a small proportion of infected subjects (2-5%) following a prolonged incubation period (15-30 years) despite a strong adaptive immune response against the virus.

Overall, these conditions offer a prospect to study the molecular basis of tumorgenicity in mammalian cells. In this review, the oncogencity of HTLV-I is being considered as an oncovirus in context of ATL.

## Introduction

*Human tumor viruses*

Oncogenecity refers to viruses that may cause cancers. Generally, the viruses that associated with malignancies are known as tumorviruses. Some researchers prefer to consider human tumor viruses as a distinct group of viruses, however, the known tumorviruses such as Hepatitis B and C viruses, Epstein-Barr virus (EBV), human herpesvirus 8 (HHV-8), Human papillomavirus (HPV), and Human T lymphotropic virus type I (HTLV-I) are various in case of families, genomes and life cycles. The aforementioned viruses are associated with malignancies such as hepatocellular carcinoma (HCC), Burkitt’s lymphoma, nasopharyngeal carcinoma (NPC), Kaposi’s sarcoma (KS), Multicentric Castleman’s Disease (MCD) and Adult T cell leukemia/lymphoma (ATL) (Table 1).

HBV is a double-stranded DNA virus of the Hepadnaviridae family which infects 350 million people worldwide. Hepatitis B can lead to liver diseases ranging from the acute hepatitis to chronic hepatitis, cirrhosis and hepatocellular carcinoma (HCC) (1-2). HCV is a positive-stranded RNA virus which belongs to the Flaviviridae family. It is estimated that approximately 180 million people are infected with hepatitis C virus worldwide (3- 4) The Epstein–Barr virus (EBV), also called human herpesvirus 4 (HHV-4), is the virus of herpes family, and is one of the most common viruses in humans. It is best known as the cause of infectious mononucleosis (glandular fever). It is also associated with particular forms of cancer, such as Hodgkin’s lymphoma, Burkitt’s lymphoma, nasopharyngeal carcinoma, and central nervous system lymphomas associated with HIV (5-7).

Kaposi’s sarcoma-associated herpesvirus (KSHV) is the most recently discovered human herpesvirus. It is the aetiologic agent of Kaposi’s sarcoma (KS), a tumor which affects more frequently on patients diagnosed with AIDS that does not receive any treatment. KSHV is also a probable cause of two lymphoproliferative diseases: multicentric Castleman’s disease and primary effusion lymphoma (8).

Human T-cell lymphotropic virus type I (HTLV-I) was the first discovered human retrovirus (9) and it has been estimated that HTLV-I infects 10-20 million people worldwide (10). This virus is endemic in several regions of the world, such as southwestern Japan, the Caribbean basin, Central Africa, South America, the Melanesian Islands and the Middle East (11- 12). The prevalence of HTLV-I infection in Iran (Mashhad) is estimated to be 2-3% of the entire population and 0.7% among blood donors (12-14). The majority of HTLV-I-infected individuals remain asymptomatic carriers (15), whereas small percentage of infected individuals develops the neoplastic disease; adult T-cell leukemia (ATL) and the inflammatory condition HTLV-I- associated myelopathy/tropical spastic paraparesis (HAM/TSP) (16). Only 5% of HTLV-I infected people develop HAM/TSP (17).

**Table 1:** A list of viruses may associated with malignancy

Virus	Virus family	Cell infected	Human malignancy
EBV	Herpesviridae	B cells, Oropharyngeal epithelial cells, Lymphoid lineage	Burkitt,s lymphoma nasopharyngeal carcinoma lymphoma
HTLV-I	Retroviridae	T cells	Adult T cell leukemia/lymphoma
HHV-8(KSHV)2	Herpesviridae	Endothelial cells, B cells	Kaposis sarcoma, PEL2 and MCD2
HBV	Hepadnaviridae	Hepatocytes	Hepatocellular carcinoma
HPV	Papovaviridae	Cervical epithelial	Cervical carcinoma
JCV	Papovaviridae	Central nervous system	Astrocytoma, glicoblastoma

**1-These tumors appear in immunosuppressed patients bear copies of EBV genomes.**

**2- KSHV: Kaposis sarcoma associated herpesvirus, PEL: Primary effusion Lymphoma, MCD: Multicentral Castleman’s disease **

**3- Although, JC virus, a close relative of SV40, is not accepted as a tumor virus and evidence of a causal role in tumor formation is lacking. more correlative evidence supports the role of JCV in the transformation of human central nervous system cells. With modificationt from : J.Butel,Carcinogenesis 21:405-426,2000**

*HTLV-I biology and pathogenesis*

*Genome structure and organization*

**Figure 1:**
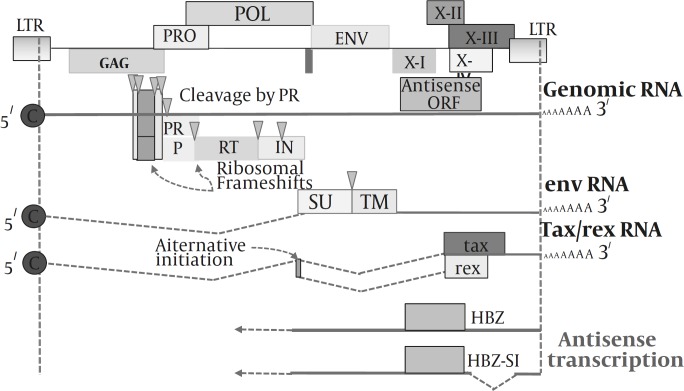
Genome organization of HTLV-I

The HTLV-I is an enveloped dimeric positive- sense single-stranded RNA virus, and like other retroviruses, the linear genome encodes the structural and enzymes such as gag, env, and pol, (Figure 1) (18). Moreover, it contains a unique region at the 3’ end, referred to the pX region, which encodes regulatory proteins, such as Tax, HBZ (for HTLV-I bZIP factor) and Rex. The biology of regulatory proteins is explained in the following sections.

The full length of HTLV-I mRNA encodes the gag protein (p55) which is then cleaved by the viral protease making the matrix (p19), capsid (p24), and nucleocapsid (p15) proteins. A sequence, from the 3 end of gag to the 5 end of pol, encodes the protease which results from ribosomal frameshifting. The HTLV-I long-terminal repeat (LTR) at both 5’ and 3’ ends of the genome contains the viral promoter and regulatory elements. In addition, the viral mRNA encodes the pol protein, single spliced mRNA encodes the env protein and a double spliced mRNA encodes the Tax and Rex regulatory proteins (19, 20).

*Tax protein*

The HTLV-I trans-activator (Tax) is a 40-kDa protein containing 353 amino acid residues located within the U3 region of the LTR (21-23). An atypical nuclear localization sequence spans the first 48 amino acids and an amino-terminal domain that interacts with the cellular transcription factors, cyclic AMP-responsive element-binding protein (CREB) and serum-response factor (SRF) (24-27). Interactions with these factors are responsible for HTLV-I LTR-trans-activation, as well as the activation of certain mitogenic genes during cellular transformation.

Tax interacts with CREB and the p300/CBP co-activator family on three 21 bp-repeats in the HTLV-I LTR and is reported to stabilize the formation of CREB/ATF-dimers bound to the DNA (28-29). The kinase-inducible domain like domain in Tax may mediate many of the interactions including the expression of many cellular genes deregulated by viral trans-activator. In fact, the competition for employing nuclear of p300/CBP may prepare Tax-dependent repression of some transcription factors, such as p53 and c-Myb (30-31). Altogether, tax ax induces or represses the expression of a large variety of cellular genes as well as regulating viral gene expression (32-33). Therefore, emerging evidence suggests that Tax serves as the primary oncogenic mediator of HTLV-I (34). Tax induces cell immortalization and transformation in vitro (35-39) as well as tumor formation in transgenic mice (40).

*Rex*

Rex is a 27-kDa phosphoprotein encoded by ORF III that localizes the nucleolus of infected cells (41-42). Rex, p27, plays a pivotal role in viral replication and the regulation of viral structural genes by functioning as a post-transcriptional regulator that increases the expression of singly spliced and unspliced viral mRNAs (env, gag, and pol, respectively) (43).

Rex is a specific RNA binding protein that binds to a cis-acting Rex responsive element (RxRE), a highly stable structure located within the R region of the viral LTR (44-45). Rex-mediated regulation is required to balance the spliced and unspliced mRNAs which is necessary for the production of infectious virus. However, the detailed mechanism(s) of Rex regulation is still highly controversial and not fully understood (46).

*p12I*

ORF I encodes a 12-kDa protein (p12I). Although, p12I expression is difficult to be displayed n HTLV-I- infected cells, some evidences have suggested its importance (20). While p12I does not seem necessary for HTLV-I replication in vitro (47-48), deletion of the acceptor splice site for the p12I mRNA results in diminishing viral infectivity in vitro (49).

This viral protein is found to have weak oncogenic activity, shares amino acid similarities with other viral oncoproteins (50), and binds to the IL-2-receptor (IL-2R) (51). p12I is necessary for the infection of primary lymphocytes in vitro (52). Therefore, p12I may have an important role in the activation of host cells at the early stages of infection where interaction of the protein with host cell signal will contribute to the host cell activation, thus affecting more viral infection.

Two variants of the p12I protein have been demonstrated: one has a Lysine at position 88 which is found in HTLV-I strains from TSP-HAM patients; the second has an Arginine at position 88 which is found in HTLV-I strains from all ATL patients and healthy carriers (53).

It has been suggested that p12I has significant roles during the early stage of HTLV-I infection establishment. Furthermore, the ability of p12I for binding to the MHC I heavy chain and making it susceptible for degradation may decrease the viral peptide MHC I- complexes on the infected cell surface and protect them from lysis by cytolytic T lymphocytes (CTLs) (54).

*Transmission of HTLV-I*

HTLV-I can infect some cell types including T cells, B cells, and synovial cells. Many of the studies demonstrated that the transmission of HTLV-I should occur by healthy infected cells and is very inefficient by free virion (REF). This is because HTLV-I transmits naturally by the cell-to-cell manner. Whilst an HTLV-I-infected cell attaches to uninfected cells, the HTLV-I-infected cells form “virological synapses” with uninfected cells (55). The receptor for HTLV-I is glucose transporter type 1 (GLUT1) (56). Its expression on T lymphocytes is enhanced by mitogens or transforming growth factor (TGF-β) (57), which has been shown to increase the infectivity of HTLV-I.

HTLV-I-infected cells in the human body are transmitted via three major routes: (1) mother-to-infant transmission (mainly breast-feeding), (2) sexual transmission, (3) parenteral transmission. It is worth noting that fresh frozen plasma from seropositive donors does not transmit HTLV-I (58). Moreover, as living cells can be eliminated by freezing and thawing, therefore, feeding the mother’s frozen breast milk to infants will not increase the risk of viral transmission (59).

*Clonal proliferation of HTLV-I-infected cells*

**Figure 2:**
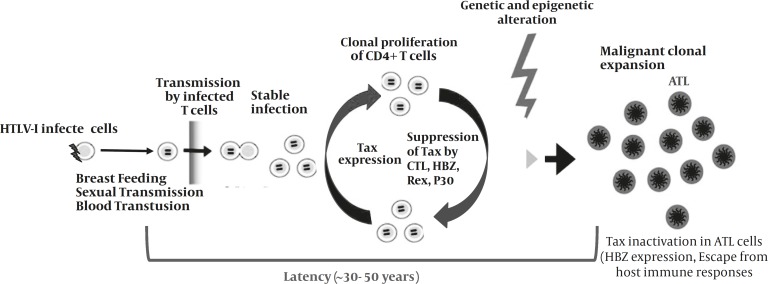
HTLV-I transmission, infection, virus-host interactions, and onset of ATL

After transmission, HTLV-I is disseminated by de novo infection and clonal proliferation of infected cells. Tax plays a central role for increasing the number of HTLV-I-infected cells by promoting proliferation and suppressing apoptosis; proliferation of infected cells seems to be oligoclonal (Figure 2) (60). A prospective study from carrier state to ATL revealed that such clonal proliferation is directly associated with the onset of ATL (61), therefore, these studies illustrate that HTLV-I-infected clones can transform to malignancy during the carrier state.

HTLV-I clonal cells are more heterogeneous and less stable during sero-positivity than in long-term carriers (62). Several factors may effect on this phenomenon including the host defense pressure by cellular immunity (63), the viral factors, particularly oncogenic proteins, and the sites of the integration.

Cell-to-cell spreading is the main rout of HTLV-I transmission. Tax mainly promotes T cell proliferation of HTLV-I infected cells. However, other HTLV-I proteins can enhance this process. Cytolytic T cells (CTLs) can prevent the proliferation by killing of Tax expressing targets cells. The expression of Tax is inactivated by several mechanisms, suggesting that Tax is not necessary in ATL stage and HBZ may be more effective. Idea from (65) with modification.

*Oncogenic function of HTLV-I proteins*

*Immortalization / transformation of T cells*

Many studies have demonstrated that HTLV-I genes (e.g. tax and HBZ), can induce various cellular dysfunctions, genetic and epigenetic alterations, and the host immune system may be involved in the leukemogenesis of ATL (64). However, there are no clear determinants that differentiate the subject develop ATL from those who remain asymptomatic.

Immortalization of lymphocytes infected by HTLV-I requires Tax, however, Tax-immortalized cells, but nontranformed cells are still IL-2 dependent. Therefore, infected cells could not pass the G1 check point without exogenous growth factors such as IL-2. In addition the inactivation of tumor suppressor gene p53 contributes to the accumulation of genetic mutations (65). Alternatively, alterations and errors are accumulated progressively by several viral proteins in the host genome during the latent period, finally leading to the onset of ATL (66).

CD4+-CD8+ cells, and immature CD4--CD8- cells from bone marrow can be transformed by HTLV-I. Transformed cells not only display an activated phenotype but also in some cases retain T-cell functional properties. Among the functional activities of HTLV-I, transformed cells have reported to be able to induce suppressor-cell activity (67) and retain antigen-specific responses and cytotoxic function (68).

*HTLV-I and apoptosis*

**Figure 3:**
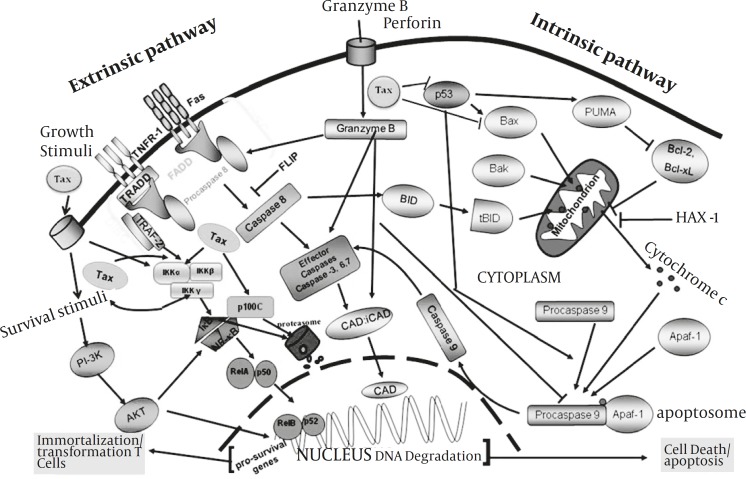
Schematic representation of pathways used by Tax to prevent programmed cell death

The programmed cell death is one of the major effectors mechanisms in NK cell and CTLs for killing the virus infected and malignant cells. The effect of HTLV-I infection on programmed cell death is less clear. Tax has been shown to repress Bax gene-expression as is shown in figure 3 (69). Since Bax promotes apoptosis by inhibiting Bcl-2, this may imply a molecular mechanism for the resistance of HTLV-I-infected T cell lines to apoptosis inducing stimuli (70). However, several reports have shown that HTLV-I-infected T cells can be induced to undergo apoptosis (69, 71). Other reports have suggested that Tax may induce apoptosis too.

With modification from Rezaee *et al*, 2006, to fit for HTLV-I. Apoptosis is one of the main mechanisms of immune system for killing virus infected or malignant cells. The extrinsic pathway of apoptosis (left side) is triggered by the binding of ligands to death-inducing membrane proteins. This signaling event leads to the recruitment of Fas-associated death receptor (FADD) which in turn activates caspase 8. Consequently, caspase 8 activates ‘effector’ caspases (caspases 3, 6 and 7) and releasing caspase-activated DNase (CAD) which in turn lead to cell death. During conditions of cellular stress, such as DNA damage and growth-factor deprivation, the intrinsic apoptosis pathway (the pathway that is activated most frequently by the tumor suppressor protein p53) is activated (right side). The intrinsic apoptotic pathway converges on the disruption of mitochondrial membranes and consequently, after some molecular activation events, results in releasing CAD. The balance of the pro- and anti-apoptotic bcl-2 family proteins determines whether apoptosis proceeds. There is overlap between the intrinsic and extrinsic apoptotic pathways, as caspase 8 can cleave and activate the bcl-2 family protein.

Therefore, manipulation of both the intrinsic and extrinsic pathways is very important for dissemination of virus to evade from NK cell and CTLs antiviral activities. The precise mechanism that HTLV-I inhibits apoptosis is unclear, but it is assumed that Tax may have the central role by suppressing p53 and BAX and stimulation of survival signals via PI3K and NF-κB pathways.

Activation of NF-κB by immune stimuli extrinsic and intrinsic pathways, which are based on degradation of IkB or processing of p100, respectively. The canonical pathway is stimulated by diverse cellular stimuli, such as antigens and cytokines, and is dependent on the trimeric IKK complex as well as certain upstream kinases, such as MEKK3 and PKCψ. The noncanonical pathway responds to a subset of TNF family members, including BAFF and CD40L, and requires NIK and its downstream kinase IKKα but not IKKβ or IKKγ. Tax activates both NF-κB pathways by physically targeting two different IKK complexes, both requiring the adaptor protein IKKγ. Formation of the noncanonical Tax/ IKK complex requires the interaction of Tax with both IKKγand p100.

Therefore, manipulation of both the intrinsic and extrinsic pathways is very important for dissemination of virus to evade from NK cell and CTLs antiviral activities. The precise mechanism that HTLV-I inhibits apoptosis is unclear, but it is assumed that Tax may have the central role by suppressing p53 and BAX and stimulation of survival signals via PI3K and NF-κB pathways.

*Proviral load*

Recently, HTLV-I proviral load levels have been evaluated as important predictors of development of ATL and HAM/TSP. Many studies show that HTLV-I proviral load are significantly higher in ATL and HAM/TSP compared to HTLV-I healthy carriers (72-75), (76-77).

 Proviruses of HTLV-I are clonally integrated in ATL patients, but randomly integrated in HAM/TSP patients. The efficiency of HTLV-I replication is shown by the proportion of PBMCs that carry HTLV-I provirus (78). Moreover, the amount of HTLV-I proviral DNA in-patients with HAM/TSP is 3-5 folds and in patients with ATL is 5-15 folds higher than HTLV-I carriers (61, 79-80). The integration of HTLV-I in PBMC of HAM/TSP patients is usually polyclonal (78). Non clonal integration has been observed in HTLV-I carriers of non-HAM/TSP family (81). Taken together, these results suggest that increased HTLV-I proviral DNA load and the presence of IgM antibody and high titres of IgG and IgA antibodies to HTLV-I proteins appear to distinguish HAM/TSP patients from HTLV-I carriers. Furthermore, high titres of HTLV-I antibody and proviral load among HTLV-I carriers maybe useful predictive markers for development of HAM/TSP (75).

Previous studies of infected human subjects suggest that high proviral load is associated with increased tendency to develop HTLV-I-associated HAM/TSP, while ATL is associated with extremely high levels of provirus (76-77, 82-83) (Akbarin *et al*, unpublished data). Counting HTLV-I infected cells in healthy carriers and ATL patients (84) determine the susceptibility of HTLV-I associated diseases (85-86) the influence of cytokines (87-88, 90). A high HTLV-I proviral load is currently considered as one of the main indicators for the progression to ATL. In a prospective study in Japan, 14 participants of asymptomatic HTLV-I carriers progressed to ATL, all of whose baseline proviral load levels were high (range, 4.17 to 28.58 copies/100 PBMCs). Therefore, the author suggested that those with a high proviral load level (∼ > 4-7 copies/100 PBMCs) are included in a high-risk group for developing ATL. Statistically analyses confirmed that a higher proviral load level was a strong factor in the development of ATL (91).

Pro-viral load measurement by Tax expression is also valuable to monitor viral activity in HTLV-I associated diseases in ATL patients and it is useful for monitoring of patients following administration of medications such as interferon-α and determination of the efficiency of medication (92-93).

*Tax*

*HTLV-I transcription by Tax*

Appropriate HTLV-Ireplication and viral gene expression are associated with sufficient expression of viral oncoprotein Tax. The mechanism by which Tax activates viral transcription is well known. For viral transcription, Tax interacts with CREB and recruits the co activator CREB binding protein (CBP), forming a nucleoprotein complex on the three viral cyclic AMP-responsive elements (CREs) in the HTLV-Ipromoter. Each three 21 bp repeats contains a cAMP response element (CRE) core. In the presence of Tax, gene expression driven by multiple copies of the 21- bp repeat element can increase up to 100-fold or higher. Cellular basic domain-leucine zipper (bZip), transcription factors-CREB, ATF-1, the 21-bp repeats, and Tax form stable ternary complexes (94,101). In these complexes, Tax binds the bZIP domains of CREB/ATF-1 (28, 102-104) which binds to the DNA of the G/C-rich sequences achieving the DNA sequence of LTR trans-activation. (94, 99, 105-106). The importance of this segment of viral DNA is well established, however, some studies have failed to identify an interaction between Tax and the DNA. In the context of these complexes, Tax recruits more transcriptional co-activators, CREB binding protein (CBP)/p300 and some other transcription factors for an appropriate gene activation (29, 94, 106-107).

*Activation of NF-κB pathway by tax*

NF-κB/Rel family of transcription factors are suppressed by I-κB proteins in cytoplasm, (108). During activation of NF-κB by extracellular stimuli such as interleukin-1(109), tumor necrosis factor-α (TNF-α), bacterial lipopolysaccharide (LPS), or by HTLV-I Tax, I-κBα and I-κBβ become serine phosphorylated by I-κB kinase (IKK). Then these inhibitory proteins are marked for rapid degradation in proteasome complex (Figure 3). Activation of NF-κB pathway is very important in proliferative disorders.

The non-canonical pathway of NF-κB activation is important for B-cell proliferation and lymphoid organogenesis and is activated in response to some inducers such as lymphotoxin β and B cell activating factor (108). Different physiological inducers of NF-κB activate either the canonical or non-canonical pathway, whereas, Tax can activate both. Activation of I-κB kinase (IKK) by Tax is due to a direct interaction between Tax and IKKγ (110-113). Tax binds directly to the 201-250 amino acid residues in IKKγ (110-114). Recent data have indicated that via a tripartite interaction, Tax, protein phosphatase 2A (PP2A) and IKKγ form a stable ternary complex. In this context, PP2A activity is inhibited or diminished (115). These results suggest that PP2A is a negative regulator of activated, phospho-IKK, and PP2A inhibition by IKKγ-bound Tax which maintains IKK in a phosphorylated and active state, causing constitutive phosphorylation and degradation of I-κB, nuclear translocation of NF-κB/Rel, and potent activation of genes under NF-κB/Rel control (Figure 3).

The classical pathway is mediated by the NF-κB-inducing kinase (NIK) and IKKα, and is independent of IKKγ (116). Phosphorylation of p100 targets p100 for ubiquitination and processing by activated NIK-IKKα complex (116-117). Tax-mediated p100 processing, however, requires both IKKα and IKKγ (112, 116, 118). Tax appears to alter p100’s conformation by direct binding to two short amino-terminal helices (αA and αβ) in p100 (119) and at the same time activate IKKγ/IKKα to facilitate p100 phosphorylation and processing (112, 116, 119). Because the non-canonical pathway is silent in T lymphocytes, its aberrant activation by Tax in T-cells may play an important role in Tax-mediated T-cell activation and transformation. On the other hand, cell transfection experiments support the importance of Tax as an intracellular NF-κB inducer, it should be noted that cells from ATL patients have elevated NF-κB activity even when Tax-expression is ultimately shutdown. This finding suggests that Tax may be used to initiate but may not need to maintain NF-κB activation (120). In fact, Higuchi *et al*. (121) have proposed that CD30 serves a role in Tax independent activation of NF-κB. CD30 is a member of the TNF receptor superfamily and interestingly is a marker of malignancy in Hodgkin’s lymphoma (122). Finally, NF- κB activation by Tax over regulates many cellular genes including those of IL-2 receptor α chain, GM-CSF, stimulatory surface receptors OX40, IL-13, IL-15, ICAM1, and anti-apoptotic proteins (111, 123-124). Role of these proteins in proinflammatory response and lymphocyte survival are obvious and are likely to be critical to the development of HAM/TSP and ATL.

*HBZ*

The complementary strand of HTLV-I proviral genome generates distinct 2.6 kb and 2.9 kb transcripts, driven by promoter in the 3’ LTR (125). Subsequently, a novel viral protein encoded by these transcripts was identified (126). This protein, designated HBZ (for HTLV-I bZIP factor), contains nuclear localizing motifs (127), and a N-terminal transcriptional activation domain and a leucine zipper motif in its C terminus (128) (Figure 1). C-terminal leucine zipper (LZ) region deletion of HBZ in an infectious proviral clone of HTLV-I had no effect on the ability of the virus to replicate and immortalize lymphocytes for growth in culture. Eliminating HBZ expression results in significant reductions in proviral load and attenuated antibody response against the viral proteins in rabbit model (129).

When expressed exogenously HBZ interferes with Tax mediated transactivation by hetrodimerizing with CREB-2 and preventing recruitment of CREB-2 to the TRE and cyclic AMP response element sites in a dose dependent manner (130), providing evidence for HBZ’s role as a potential negative regulator of Tax-mediated viral transactivation is important.

Other cellular transcriptional factors appear to be the target of HBZ functions. HBZ interacts with Jun-D and stimulates its transcriptional activity (131) while suppressing transactivation by c-Jun (132). HBZ represses AP-1 transcriptional factor activity by impairing both the DNA-binding ability and the stability of c-Jun protein (133). These studies provide evidence for possible alteration of cellular processes by HBZ following HTLV-I infection.

*ATL*

ATL is the most common outcome of infection with HTLV-I, and in areas of endemicity, it occurs in 2-5% of HTLV-I-infected individuals (134). It was reported that the median age of onset of ATL is 56 years (135), suggesting a long period of latent infection (134). It has led to the assumption that most, if not all, cases of ATL develop in individuals infected with HTLV-I since birth (136).

ATL can be presented in one of several forms (which may be stages in the natural history of ATL). Although there are significant geographic variations in the contribution of these forms in the initial presentation, the most common presentation is acute leukemia.

The most benign detectable form of ATL is an asymptomatic pre-leukemic phase which is usually diagnosed incidentally when the examination of a peripheral blood smear reveals abnormal lymphocytes with characteristics of lobulated nuclei (flower cells, Figure 2) (137).

Smoldering ATL is the most benign symptomatic form of disease and is characterized by cutaneous but not visceral lesions, a normal peripheral blood leukocyte count, and a few circulating leukemic cells. The development of chronic ATL is marked by visceral involvement and is evidenced by lymphadenopathy, hepatosplenomegaly, and peripheral blood leukocytosis. However, both of the relatively benign ATL can progress to acute ATL.

The median life expectancy of individuals with acute ATL is 11 months. Patients with acute ATL have cutaneous and visceral involvement, peripheral blood leukocytosis, elevated levels of lactate dehydrogenase and bilirubin, and often hypercalcemia. ATL is associated with severe immunosuppression as evidenced by the susceptibility of these patients to various opportunistic infections, including Pneumocystis carinii pneumonia, cryptococcal meningitis, candidal esophagitis, and disseminated cytomegalovirus infection (138). Occasional long-term survival has been described, but the disease is usually unresponsive to conventional chemotherapy. Recent reports of immunotherapeutic trials allow for a degree of optimism (139).

ATL is generally resistant to the chemotherapy and carries a dismal prognosis particularly for the acute and lymphoma subtypes. Promising results were obtained regarding the combination of zidovudine and interferon-alpha. Chronic ATL has a relatively better outcome, but poor long-term survival is noted when patients are managed by a watchful-waiting policy or by chemotherapy. In ATL cell lines, arsenic trioxide shuts off constitutive NF-kappa B activation and potentiates interferon-alpha apoptotic effects through proteasomal degradation of Tax. Clinically, arsenic/interferon therapy exhibits some efficacy in refractory aggressive ATL patients. The aforementioned data were leading us to investigate the efficacy and safety of the combination therapy of arsenic, interferon-alpha, and zidovudine in 10 newly-diagnosed chronic ATL patients. An impressive 100% response rate was observed including 7 complete remissions, 2 complete remissions but with more than 5% circulating atypical lymphocytes, and 1 partial response. In conclusion, treatment of chronic ATL with arsenic, interferon-alpha, and zidovudine is feasible. Overall, the clinical trial data strengthen the concept of oncogene-targeted cancer therapy (139) and a long-term follow up will clarify whether this will end to disease cure.

*HTLV-I proteins in ATL development*

The pX region of the HTLV-I genome encodes a number of nonstructural proteins, including Tax(140), Rex (141), and the accessory proteins encoded by the open reading frames I (p12I and p27I) and II (p13II and p30II) (Figure 1). The most notable viral regulatory protein is Tax (34,140, 142,).

Although the basis of cellular transformation by Tax is not fully understood (143-144); (145), Some studies have demonstrated that Tax is the major transforming protein of HTLV-I, However, no common integration site is found among patients, integration of the proviral genome into host cell DNA is monoclonal in transformed cells, suggesting that integration occurs prior to the transformation.

The tax gene plays a central role by its pleiotropic actions in the proliferation and leukemogenesis of HTLV-I-infected cells in vivo (34). However, its transcription is detected in only 34% of ATL cases (146). Tax production is impaired by several mechanisms: (1) - genetic changes of tax gene (146), (2) - deletion of 5’-long terminal repeat (LTR) (147), and (3) - DNA methylation of 5’ LTR (146, 148). The frequency of defective provirus, particularly type 2, was much higher in acute and lymphoma-type ATL than in chronic type ATL, suggesting a close association with disease progression. Tax expression is absent or severely impaired in ATL cells with heavily methylated 5’-LTR. Such changes are predominantly observed in aggressive subtypes of ATL, which suggests that at this stage, ATL cells acquire the ability to proliferate without Tax expression; such changes enable ATL cells to escape from the host immune system because tax is the most immune-dominant protein (Figure 1 and 5). This finding reveals a duality in the Tax protein: its expression induces proliferation and inhibits apoptosis of HTLV-I-infected cells, and evokes the host’s immune response including cytotoxic T cells to kill virus-infected cells. Therefore, after dissemination of virus and during latency, loosing tax protein is a pivotal mechanism for HTLV-I to escape from host immune system.

Although Tax lacks a cellular homologue (149), it functions like an oncogene, inducing leukemogenesis by causing aberrant transactivation of cellular growth regulatory genes. A large group of cellular genes involved in T-cell growth are activated by Tax including interleukin 2 (IL-2), the high-affinity subunit of the IL-2 receptor (IL-2Ra), IL-3, IL-4, granulocyte–macrophage colony-stimulating factor, proliferating cell nuclear antigen, TGF-β, vimentin, proenkephalin, egr-1, egr-2, fos, c-myc, bcl-xL, and Jun (150).

The mechanisms by which Tax modulates viral and host cell transcription factors toward transformation are of considerable interest. Altogether, the studies suggest that Tax may initiate a cascade of events leading toward transformation that, at later stages, may not require or must be eliminated due to immunogenicity for the host immune response (Figure 5).

Tax-mediated immortalization of HTLV-I-infected T cells toward leukemogenesis by genetic and epigenetic changes in provirus and host genes. However, HTLV-I gene expression also can be recognized by T cells result in activation of host immune system which eliminates Tax-expressing target cells. Therefore, the host immune system pressure in such circumstances is in favor of proliferation of Tax negative HTLV-I infected cells.

*Activation of the PI3 kinase pathway and ATL*

Some studies have reported constitutive activation of the phosphoinositide 3-kinase (PI3K) pathway as a common characteristic of HTLV-I- and Tax-transformed cells (Figure 2). Treatment of IL-2-independent HTLV-I transformed T-cells with inhibitors of PI3 kinase leads to a p27KIP1- dependent cell cycle arrest (151).

*Somatic changes in ATL cells*

Mutation of p53, and deletion of p16 have been reported in ATL with a poor prognosis (152).Therefore; these genetic changes are believed to be associated with the disease progression. A transcriptional profile of ATL cells by DNA chip analysis identified aberrantly transcribed genes (153). Among them, the expression of tumor suppressor in lung cancer 1 gene is overregulated in ATL cells (154). However, its ectopic expression is associated with leukemogenesis possibly due to conferring an adhesive phenotype to ATL cells.

**Figure 4:**
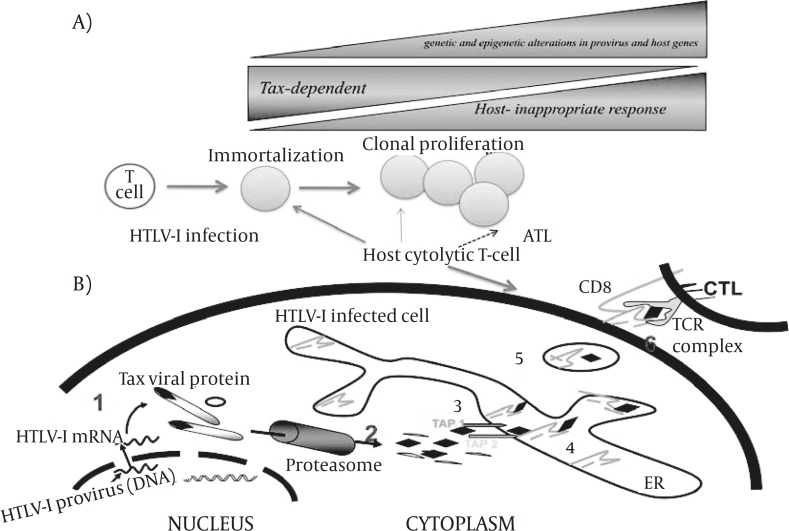
A schematic illustration of HTLV-I oncogenesis, HTLV-I infection, host CTLs responses and onset of ATL

Epigenetic changes are recognized as mechanisms implicated in oncogenesis as well as genetic changes. Since genetic changes in specific genes in ATL cells have not been identified except for p53 and p16, and there is no consistent chromosomal change, it is possible that epigenetic changes such as DNA methylation (155) play an important role in leukemogenesis by inhibiting the transcription of tumor suppressor genes or inducing aberrant expression of oncogenes. On the other hand, EGR3 gene has been demonstrated to be hypermethylated in ATL cells (156). EGR3 is a transcriptional factor, which is essential for transcription of the FasL gene (157). Normal activated T lymphocytes express FasL as well as Fas antigen. Apoptosis induced by autocrine mechanisms is designated activation-induced cell death and controls the immune response (158). Although ATL cells express Fas antigen, they do not produce FasL, thereby enable ATL cells to escape from programmed cell death. Thus, epigenetic changes of the host cell may play a pivotal role in oncogenesis of ATL.

*Tax protein and cell cycle deregulation*

Several lines of evidence indicate that p40tax is the oncogene responsible for viral lymphocyte-transforming and leukemogenic properties (38). Mechanistically, several biochemical features of the protein can cooperate to transform, in which transcriptional stimulation of cellular signal transducers, cytokines (159) and anti-apoptotic effectors exist. Tax has capacity to stimulate aneuploidy and interfere with DNA repair (160), which could indirectly support malignant progression. A major mechanistic explanation for the mitogenic and immortalizing effects of the Tax oncoprotein is provided by its ability to stimulate the G1- to S-phase transition in T-cells (161-163).

In mammalian cells, G1-progression is controlled by the sequential activation of several cyclins and cyclin-dependent kinases (CDKs), starting with CDK4, CDK6 and CDK2. Tax activates CDK4, CDK6 and CDK2 leading to phosphorylation of retinoblastoma (Rb) tumor suppressor proteins and liberation of the transcription factor E2F (161, 164). Moreover, Tax may induce Rb degradation (165) and increase cellular E2F synthesis (166-167). Several indirect effects of Tax and features of HTLV-infected cells may support the impact of Tax on CDK.

In general, Tax shortens the length of G1 and accelerates entry into S phase (150, 168). In Tax-expressing cells and ATL cells in culture, Tax stimulates cyclin/cdk activity; resulting in hyperphosphorylated Rb. Tax also increases E2F transcription and protein levels (164,167,169).The cumulative effect of hyperphosphorylated Rb and increased E2F transcription cause the availability of more active E2F, which facilitates the entry of Tax-expressing cells into S phase. Tax also increases the levels and activity of several cyclin/cdk partners, which are important in G1 progression and S phase entry.

 Cyclin D2 expression is also overregulated by interleukin-2 receptor (IL2-R) signals (170- 171). Tax may cooperate with interleukin-2 (IL-2) signaling either indirectly, through stimulating the expression of IL-2Rα or directly, by activating the cyclin D2 promoter (172- 173). Tax also represses the function of distinct tumor suppressor proteins which interfere with G1- to S-phase transition. (174). The interaction of the cyclin D/CDK components provides a major explanation for the G1-phase stimulating effects of Tax. Direct association with Tax enhances CDK4 activity. This increased kinase activity in the presence of Tax may be explained by intensified association of CDK4 and its positive cyclin regulatory subunit and resistance of the complex to inhibition by p21CIP1(175).

The 40 N-terminal amino acids of Tax are sufficient to bind cyclin D2 and CDK4. Within CDK4 a N- and a C-terminal domain are relevant for Tax binding. Taken together, these findings suggest that Tax stimulates G1- to S-phase transition by supporting the association of CDK4 and cyclin D2. Furthermore, these studies support the conclusion that CDK4 activity is stimulated through conformational changes of the enzyme directly mediated by Tax (176).

 Cell cycles checkpoints are activate in DNA damage. Tax disrupts the DNA damage-induced G1/S checkpoint (controlled by cyclin E/cdk 2), in part by inactivation of p53. Normally when DNA damage is detected during G1, p53 activates the cdk inhibitor p21waf1, which in turn, binds and inactivates cdk2. Although p53 is inactivated, Tax-expressing cells have higher levels of p21waf1 because Tax transactivates the p21waf1 promoter (177-178); (164). Loss of p53 function prevents proper G1/S arrest, p53-mediated apoptosis, and DNA repair, all of which contribute to cellular transformation. A series of studies have demonstrated that in HTLVI-infected cells, Tax has the ability to abrogate the transactivating function of p53 (Figure 2). Interestingly, whereas p53 mRNA levels remain unaffected in these cells, p53 protein levels are elevated, implying that Tax represses p53 function by increasing protein stability and/or through post-translational modifications (179).There are conflicting opinions on the mechanism by which Tax mediates the loss of p53 function in vivo. Although Tax does not bind to p53, it alters its subcellular localization, or disrupts its DNA-binding activity (71, 180-181).

However, more studies should be conducted to reach more reasonable mechanisms to understand how HTLV-I can pass the cell cycle checkpoints and push the cell toward mitosis and proliferation leading to cell transformation. 

T-lymphocyte activation is a way for clonal expansion in the immune system for encountering pathogens and is highly regulated. HTLV-I Tax has developed numerous mechanisms to subvert this control and induce cell cycle promotion in the absence of physiological signals. For instance, high levels of IL-2 are required to promote the progression of resting T cells through the cell cycle. After binding to its receptor (IL-2R), IL-2 triggers its production of as well as IL-2Ra. Cells expressing the high-affinity IL-2R are more sensitive to IL-2 and continue to proliferate (182). In first stage of HTLV-I infection, most infected T cells require IL-2 to proliferate; however, later in infection, their proliferation is IL-2 independent (183). Tax has been shown to transactivate both the IL-2 and IL-2Ra promoters through NF-κB-like signaling pathway (184-186). Moreover, IL-2-independent HTLV-I-infected T cells have a constitutively activated JAK-STAT pathway. As a result, these cells no longer require IL-2 to activate the pathway and proliferate. Conclusively, HTLV-I infected T cells that still require IL-2 to proliferate do not have a constitutively activated JAK-STAT pathway (187-188). 

ATL cells secrete an increased amount of TGF-β because of Tax transactivation of the TGF-β promoter (189-190); however, the cells are resistant to growth inhibition by TGF-β (191). This resistance occurs due to the numerous effects of Tax on the TGF-β pathway.

Although Tax enhances TGF-β production, infected cells are resistant to TGF- β growth inhibition because Tax disrupts the TGF- β signaling pathway and interferes with the function of growth inhibiting proteins induced by TGF-β.

An additional disruptive effect of Tax emanates from its impact on the ability of the cell-cycle machinery to regulate DNA replication and cell division (142,192-194).

Despite the fact that nearly 70% of all cancers demonstrate aneuploidy (including both ATL and HTLV-I-infected cells), only rarely have genetic defects which are identified in mitotic checkpoint genes, implying that other events must alter function of the mitotic spindle checkpoint (MSC) (195-196). Research on both DNA repair and MSC are vital to understand HTLV-I-mediated cellular transformation and are highly prior for future studies.

*Activation of NF-κB by HTLV-I*

*NF-κB activation by immune stimuli*

The NF-κB proteins are normally sequestered in the cytoplasm by physical interaction with a family of inhibitory proteins, including IkBa, IkBb, and related proteins (197). The NF-κB precursor proteins, p105 and p100, contain IkB-like sequences in their C-terminal portion and also function as NF-κB inhibitors (198). Thus, the processing of these precursor proteins serves to both generate mature NF-κB subunits and disrupt their IkB-like function.

The latent NF-κB complexes can be activated by diverse immune stimuli, such as antigens, cytokines, and microbial components, which target two alternative NFkB signaling pathways: the canonical and noncanonical pathways (108, 199).

*Aberrant activation of NF-κB by HTLV-I*

Under normal conditions, the signals mediating NF-κB activation in T cells as well as most other cell types are transient and stimulate predominantly the canonical NF-κB pathway. Such a signaling mechanism ensures the rapid, but short-lived, nuclear expression of NF-κB members that are required for temporal proliferation and survival of antigen-stimulated T cells. This mechanism is achieved through different levels of regulation. First, following stimulation by an antigen, both the T cell receptor (TCR) and its proximal signaling molecules are downregulated, thus preventing persistent signaling through the cell surface receptor (200). Second, the NF-κB signaling pathway involves a negative feedback mechanism, whereby the activated NF-κB induces the expression and de novo synthesis of the inhibitory protein IkBa (201-202). The newly synthesized IkBa is able to enter the nucleus and stop the function of NF-κB. Despite its tight control in normal T cells, NF-κB is constitutively activated in both HTLV-I-transformed T-cell lines and freshly isolated ATL cells (186, 203-205). The persistent activation of NF-κB by HTLV-I appears to be mediated through a mechanism that does not involve the TCR or its proximal signaling molecules, such as Src and Syk families of protein tyrosine kinases (PTKs). In fact, a characteristic of HTLV-I-transformed T cells is the loss of TCR and upstream PTKs (183, 206-207,). It is generally believed that the viral Tax protein serves as an intracellular NF-κB inducer that acts by bypassing the TCR-proximal signaling factors.

A main point of Tax-stimulated NF-κB activation is the marked induction of nfkb2 gene product, p52, as well as the canonical NF-κB members (208-209) (Figure 3). In normal T cells, p52 exists largely as its precursor, p100, even when the cells are activated by T-cell mitogens (209). When human T cells are infected with HTLV-I, p100 undergoes active processing, a phenotype that is also detected in a large panel of HTLV-I-infected T-cell lines (209) and leukemic cell-derived ATL cell lines (210).

Over the past decades, significant progress has been achieved through understanding the mechanism of NF-κB activation by Tax. Indeed, Tax binds to several NF-κB members, including RelA, p50, and p52 (26, 211). Tax also interacts with members of the IkB family, such as IkBa, and the NF-κB precursor proteins p105 and p100 (208, 212). While such virus/host interactions may contribute to the activation of NF-κB by Tax, it is, however, clear that Tax cannot directly activate NF-κB via physical interactions with NF-κB or IkB members. Strong evidence suggests the requirement of the cellular protein kinase IKK in Tax mediated NF-κB activation.

Some studies revealed that Tax induces the degradation of both IkBa and another IkB member, IkBb (213-214). Since Tax has no kinase activity, these findings argued for the activation of a cellular IKK by Tax. More direct evidence for the involvement of a cellular kinase in Tax-mediated NF-κB activation came from the finding that Tax induces IkBa phosphorylation at two regulatory serines such as serine-32 and serine-36 (215), which also serves as the sites of IkBa phosphorylation induced by cellular stimuli (216). Constitutive IKK activity was detected in both HTLV-I-infected and Tax-transfected cells. An essential role for IKK in Tax-mediated NF-κB activation was subsequently confirmed by genetic studies using both non-lymphoid and T-cell systems (217).

In addition to mediating IkB degradation and nuclear translocation of NF-κB, IKK also regulates phosphorylation of the RelA subunit of NF-κB, a modification that is required for the transactivation function of NFkB. Although IKKβ is essential for Tax-induced nuclear translocation of the canonical NF-κB (218α

*Implications of NF-κB in HTLV-I-induced T-cell transformation*

Through activation of NF-κB, Tax induces the expression of various NF-κB target genes that promote cell growth and survival as well as suppressing the expression of target genes of p53 involved in DNA repair and cell cycle checkpoint regulation. 

Activation of NF-κB is also essential for Tax-induced IL-2-independent T-cell growth (219). Loss of viral gene expression, particularly Tax in the late stages of ATL, has been shown by studies which represented the expression of the viral proteins and was barely detectable in the peripheral blood lymphocytes freshly isolated from ATL patients (220, 221).

This could partly be explained by the epigenetic changes like hypermethylation (Koiwa *et al* 2002) or defect (147) in the 5’ LTR of the HTLV-I provirus. In addition, nonsense or missense mutations of the tax gene were reported in certain ATL cases (222).

The frequent lack of detectable viral gene expression in ATL cells is thought to be a result of the host immune surveillance. Tax is essential for immortalization of infected T cells (35, 223); however, it is also known as a major target of cytolitic T-lymphocyte mediated immunity (224-225) for eliminating HTLV-I infected cells. This process may facilitate selective outgrowth of cells that lost viral gene expression and in turn acquired Tax-independent growth advantages through alterations of host gene expression (Figure 5).

## Conclusion

Investigating virus and host interaction lead us to a better understanding of molecular mechanisms of viral protein and host cellular response in pathogenesis of infection or dissemination of virus. In case of HTLV-I, such cellular and viral protein interactions pave the way for discovery of new classes of cellular modulators, which may induce cell cycle deregulation and disrupting host immune responses toward malignancy and autoimmunity.

Conducting molecular research for better understanding of molecular behavior of HTLV-I can assist the researcher to find selective therapeutic agents for malignancy and autoimmunity. With regard to the oncogenesis of Tax and its impact on survival signaling pathways such as NFkB and PI3K-Akt pathways, new therapeutic opportunities for ATL has been presented in two collaborative studies (139, 226). Moreover, new therapeutic approach is in progress in our group on HAM/TSP patients based on the result of an IFN-α study (227) and with regard to epigenetic studies (Moshfegh, *et al*,).

Based on the molecular interactions between HTLV-I and host, there are more hopes that these activities have evolved under selective pressure to become highly specific for malignancy (ATL) and Autoimmunity (HAM/TSP).
